# Twigs of dove tree in high-latitude region tend to increase biomass accumulation in vegetative organs but decrease it in reproductive organs

**DOI:** 10.3389/fpls.2022.1088955

**Published:** 2023-01-13

**Authors:** Zhengchuan Liang, Tingting Liu, Xiaoyan Chen, Wenjuan Xu, Tingfa Dong, Qinsong Liu, Xiao Xu

**Affiliations:** ^1^ College of Life Science, China West Normal University, Nanchong, Sichuan, China; ^2^ Scientific Research and Testing Unit, Sichuan Mabian Dafengding National Nature Reserve Protection Center, Leshan, Sichuan, China; ^3^ Key Laboratory of Southwest China Wildlife Resources Conservation (China West Normal University), Ministry of Education, Nanchong, Sichuan, China

**Keywords:** biomass, *Davidia involucrata*, functional adaptation, latitude, twig components

## Abstract

Adaptive traits are an important dimension for studying the interactions between rare plants and environment. Although the endangered mechanism of rare plants has been reported in many studies, how their twigs adapt to heterogeneous environments associated with latitude is still poorly known. Dove tree (*Davidia involucrata* Baill.), a monotypic rare species in China, was employed as a model species in our study, and the differences in functional traits, growth relationships and resource allocation among components of annual twig were investigated in three latitudinal regions (32°19′ N, 30°08′ and 27°55′) in the Sichuan, Southwest China. Compared with low- and middle-latitude regions, the twig diameter in high-latitude region decreased by 36% and 26%, and dry mass decreased by 32% and 35%, respectively. Moreover, there existed an allometric growth between flower mass and stem mass or leaf mass in high-latitude region but an isometric growth in low- and middle-latitude regions. At the flower level, an isometric growth between bract area and flower stalk mass was detected among in three latitudinal regions, and the flower stalk mass in the low-latitude region was higher than in the middle- and high-latitude regions for a given bract area and flower mass. At the leaf level, the growth rate of petiole mass was significantly higher than those of leaf area, lamina mass and leaf mass among three latitudinal regions, and the petiole mass in the low-latitude region was higher than in the other two regions for a given leaf mass. Our research demonstrated that the twigs of dove tree in high-latitude region tend to become smaller, and resource input increase in stems and leaves but decrease in flowers, which reflects that dove tree can adapt to the environmental changes across different latitudes by adjusting phenotypic traits growth and biomass allocation of twigs.

## 1 Introduction

The mutual relationship between plants and the environment is one of the research focuses in ecology. In many environmental factors, changes in latitude can significantly influence plants’ growth and reproduction. Due to changes in latitudes, the ambient temperature, rainfall, soil conditions, solar radiation and abundance of pollinators also change accordingly (Li et al., 1998; Silva and Eguiarte, 2003; Shi et al., 2019; Shi et al., 2020a), resulting in significant differences in individual size, resource allocation, flower morphology, as well as reproduction strategy of plants (Mcintosh, 2002; Herrera et al., 2006; Cosacov et al., 2014). These phenomena reflect the response of plants to environmental heterogeneity by phenotypic plasticity. Moreover, some studies reported that the interaction between plants and environmental factors could change the biomass allocation and the investment-benefit-cost relationship of plants, which may be a trade-off between various traits of plant organs (Liu M. D. et al., 2020; Shi et al., 2020b; Li et al., 2021; Shi et al., 2022). The trade-off mean that an organism gains benefits from one trait, another trait pays appropriate costs. This evolutionary problem faced by all plants living in a given environment is how to allocate their limited resources to maximize their survival and reproduction possibilities (Stearns, 1989). Plants can change the investment cost between different organs or within the level of organ through trade-off relationship to allocate the limited resources reasonably and achieve the optimal return to adapt the needs of different growth and development stages. Such a trade-off relationship in turn may influence morphological construction, nutrient adsorption and transportation of plants, which reflect the ecological strategy of plants in the evolution of life histories (Sun et al., 2006; Dai et al., 2020).

In addition, as the most active part of the plant branch system, annual twigs are an important channel for water and organic transportation in plants. They support the growth and development of leaves, flowers and fruits, and their biomass allocation among organs will directly affects the growth and reproduction of the plant. Therefore, it is important to study the biomass allocation and configuration characteristics in twigs for plant life history strategy (Osada, 2006). As far as the component of a twig is concerned, leaf is the important organ for plants to acquire resources. Their morphological characteristics and biomass allocation influence the capacity of carbon acquisition (Givnish and Vermeij, 1976). Stem serve as an important structural unit of plants, transporting nutrients and providing mechanical support, they also can adjust branch and leave distribution patterns in the canopy to improve photosynthetic efficiency by changing their lengths, diameters and arrangement modes (Day et al., 2002; Thomas and Winner, 2002). As a vital functional organ for the reproduction of angiosperms, flower plays an important role in attracting pollinators and in the accurate coupling of pollinators (Alexandersson and Johnson, 2002; Herrera, 2005). Since functional traits of plants are often in harmony with each other, plants have to properly allocate biomass and nutrients among different structures and functions for survival and reproduction, such as the balance among stems, leaves, flowers, and other organs, which is an important biological characteristic for plants to coordinate growth (Normand et al., 2008; Campbell et al., 2011). For example, stem and leaf present isometric growth relationship and the same proportional biomass allocation pattern holds across extant seed plant species (Niklas and Enquist, 2002). The size of branches and leaves of woody plants exhibit an isometric growth relationship in temperate forest community (Sun et al., 2006). Stem mass and leaf mass of twigs present an isometric scaling relationship in plant *Populus cathayana* (Yang et al., 2015). The size and weight of bract of *D. involucrata* population in low-altitude areas are significantly higher than those in high-altitude areas, but the latter prefers to allocate more resources in supporting structures (Liu T. T. et al., 2020). Nevertheless, the above studies mainly focused on the stem-leaf relationship or reproduction strategies, but the growth relationships between functional traits and resource allocation patterns among reproductive organ (flower), support organ (stem) and vegetative organ (leaf) at the level of twig in different latitudinal regions were rarely involved.

*Davidia involucrata* Baill. belongs to *Davidiaceae*. It is a Tertiary relict deciduous trees in China and has been listed in China Red Data Book as a Class I species for protection (Fu and Jin, 1992). As climatic changes intensify in recent decades, the habitat conditions of *D. involucrata* have changed accordingly, which resulting in a sharp decrease in natural population size (Tang et al., 2017). Therefore, it is very important to pay attention to the variations in functional traits and the relationships between resource allocation and heterogeneous environments in *D. involucrata* plants. However, previous studies on *D. involucrata* mainly focus on reproductive capability, population dynamics, community characteristics, molecular biology and physiological ecology (Li et al., 2016; Liu et al., 2019; He et al., 2020; Yang et al., 2020; Liu et al., 2021). Since changes in latitude may lead to corresponding changes in the temperature, water and light intensity in plant habitats (Osada et al., 2015), we speculated that such changes might influence functional traits and biomass allocation pattern at the twig level, especially in rare endangered plant groups with poor habitat adaptability. To test this hypothesis, three natural *D. involucrata* populations with similar altitude which located at Pingwu (PW; 104°32′ E, 32°19′ N), Tianquan (TQ; 102°26′ E, 30°08′ N) and Gongxian county (GX; 104°52′ E, 27°55′ N) in Sichuan, Southwest China, were chosen as representative groups in high, middle and low-latitude region, respectively. The functional traits (leaf, flower and stem) of annual *D. involucrata* twig were measured. Based on these findings, the growth relationships between traits were analysed with the standardized major axes (SMA) to determine whether latitude may influence the functional traits and resource allocation strategies in *D. involucrata* populations.

## 2 Materials and methods

### 2.1 Study sites

**Figure 1:**
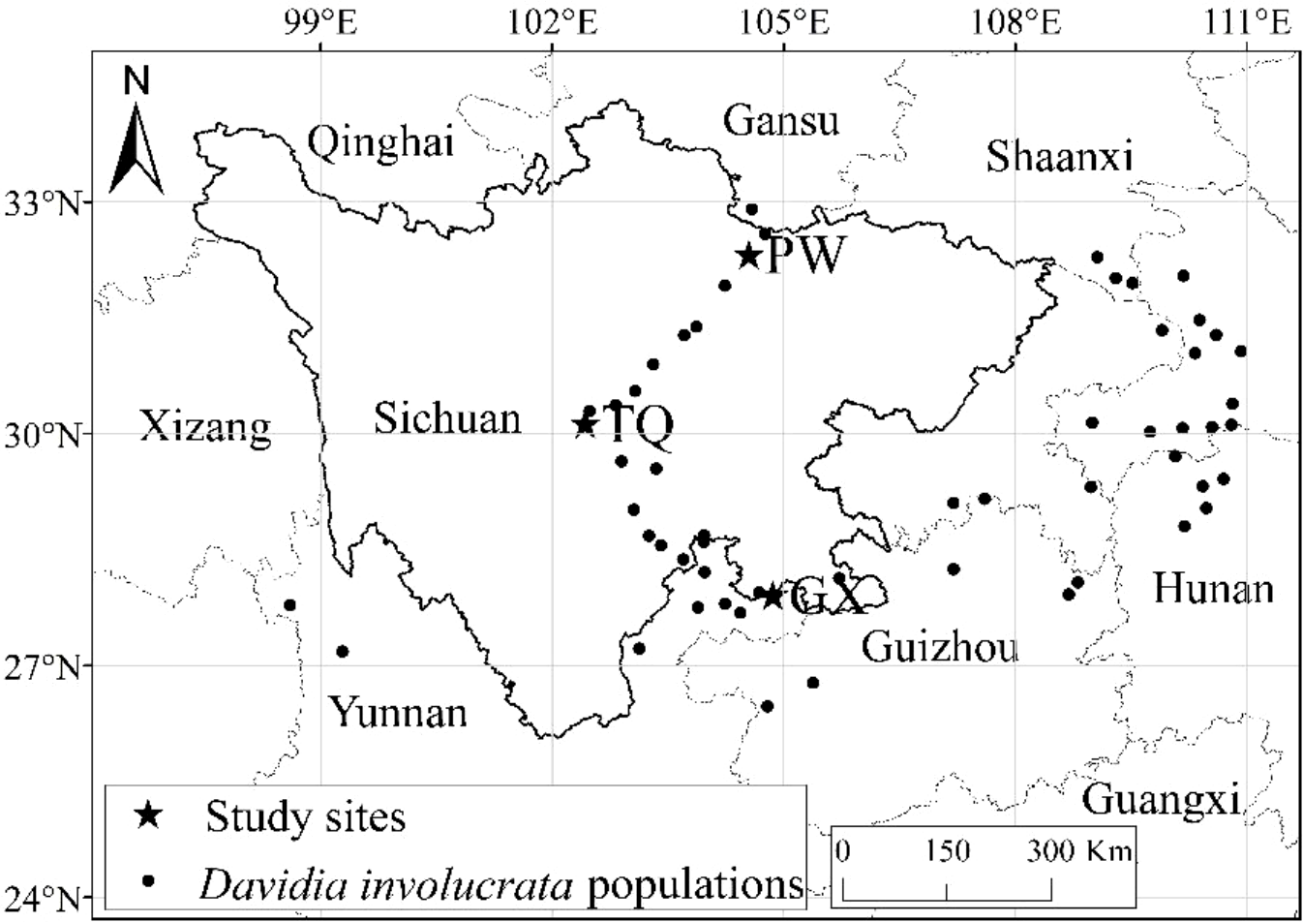
Location of study site in Sichuan, China.

Natural *D. involucrata* populations located at three latitude regions in Sichuan Province were chosen as the research objects (**Figure 1**). The geological distribution is PW, TQ and GX from north to south. All three regions are located at the intersection zone between the southeast edges of the Qinghai-Tibet Plateau and the Sichuan Basin. The soil of three sampling sites is yellow-brown forest soil, with pH value and organic matter of 6.6 and 2.66%, respectively (Chen et al., 1992). They all belong to subtropical monsoon climates with a latitude gap of about 2°, which reflects an excellent zonal characteristic of latitude. General information about the basic environment is shown in **Table 1**.

**Table 1:** The general information of *D*. *involucrata* populations in three latitude regions.

Population	PW	TQ	GX
Site	Pingwu	Tianquan	Gongxian
Longitude	104°32′ E	102°26′ E	104°52′ E
Latitude	32°19′ N	30°08′ N	27°55′ N
Altitude	1600 m	1803 m	1600 m
Mean annual temperature	10.1°C	15.1°C	18.2°C
Mean annual precipitation	866.5 mm	1735.6 mm	1126.0 mm
Soil type	Yellow-brown forest soil	Yellow-brown forest soil	Yellow-brown forest soil
Slop	40°	40°	45°
Aspect	Northeast	West	North
Forest type	Deciduous broad-leaved and Evergreen broad-leaved mixed forest	Deciduous broad-leaved forest	Deciduous broad-leaved and Evergreen broad-leaved mixed forest
Main woody species	Macrophanerophytes: *Davidia involucrata*, *Carya cathayensis*, *Quercus aliena*, *Cornus controversa*, *Machilus nanmu* Frutex: *Viburnum dilatatum*, *Litsea pungens*, *Cornus officinalis*	Macrophanerophytes: *Davidia involucrata*	Macrophanerophytes: *Davidia involucrata*, *Fagus longipetiolata*, *Sassafras tzumu*, *Acer pictum*, *Rehderodendron macrocarpum* Frutex : *Ternstroemia gymnanther*a, *Eurya loquaiana*, *Litsea pungens*,
Dominant species	*Davidia involucrata*	*Davidia involucrata*	*Davidia involucrata*
Population size	40 – 50 ha	1000 – 1100 ha	50 – 65 ha

PW, Pingwu; TQ, Tianquan; GX, Gongxian.

### 2.2 Plant sampling

In the flowering stage of *D. involucrata* populations from mid-April to early May, 6 − 8 healthy plants with similar DBH (diameter at breast height) and no insect pests were randomly selected from the three natural populations in PW, TQ and GX. Selected trees were (a) healthy with similar habitats (soil type, slope and aspect, in **Table 1**); (b) full grown with similar canopy size; (c) not to be located near the edge of the forest, or next to previously sampled trees. Then, to avoid growth differences derived from light intensity, 3 − 5 annual twigs (including stem and all leaves and flowers above the stem) were randomly collected from the sunny side of the lower crown of each plant. The range of mean DBH and mean crown was from 32.01 to 37.50 cm and from 8.55 to 10.93 m, respectively, and no significant differences were detected among three sampling sites (*P* = 0.275 and = 0.207, respectively). The plant samples were collected in sealed bags and numbered before being placed in an incubator with ice bags. They were brought back to the laboratory and stored in a 4°C refrigerator.

### 2.3 Measurement of twig traits

The morphological and biomass traits of leaf, stem and flower from each twig were measured. Lamina, petiole, bract and flower stalk were scanned using CanoScan LiDE210, and then the length, width and area of lamina and bract, as well as the petiole length and flower stalk length were measured by the software Image J version 1.47v (National Institutes of Health, USA). The diameter of stem and flower stalk were measured with an electronic vernier calliper (0.01 mm), respectively. Biomass samples were oven-dried to constant mass (70°C, 72 h), then the lamina mass, petiole mass, bract mass, receptacle mass, capitulum mass, flower stalk mass and stem mass for each twig were separately measured. The leaf area or leaf mass was the sum of lamina area and petiole area, or lamina mass and petiole mass. The flower mass was the sum of mass of bract, receptacle, capitulum and flower stalk. The twig mass was the sum of the mass of stem, leaves and flowers. The mean of leaf area, lamina length and lamina width was calculated as the total area, length or width divided by leaf number for each twig, respectively.

### 2.4 Data analysis

Statistical analyses were performed using SPSS version 25.0 (SPSS, Inc., Chicago, IL, USA). Data were checked for normality and the homogeneity of variances, and log-transformed to correct deviations from these assumptions when needed. The Shapiro-Wilk normality test was used to evaluate normal distribution. One-way ANOVAs were used to determine differences among three latitudinal regions, and the Least Significant Difference (LSD) test was employed to detect possible differences among means. Differences were considered significant if the *P* < 0.05. The growth relationships between traits were analysed with a type II regression model, and the standardized major axes (SMA) were performed using the SMATR Version 2.0 (Falster et al., 2006). Confidence intervals for individual regression slopes were calculated according to Pitman (1939), and tests for heterogeneity of regression slopes between the sexes and calculation of common slopes where homogeneity of slopes were demonstrated followed Warton and Weber (2002).

## 3 Results

### 3.1 Comparisons of the traits of twig components in three latitude regions

The morphological traits of the reproductive organ, support organ and nutritive organ of *D. involucrata* twig were significantly different among different latitude regions. Stem diameter and flower stalk diameter decreased significantly with increasing latitude (*P* < 0.001 and < 0.001, respectively). The bract area and flower stalk length were the highest in low-latitude region, and the bract area showing significant differences from that in the middle- and high-latitude regions, and the flower stalk length was significantly different from that in the middle-latitude region (*P* = 0.014 and = 0.005, respectively). Moreover, the mean leaf area, mean lamina length and mean lamina width in high-latitude region were significantly higher than those in the other two regions (*P* < 0.001 and < 0.001 and < 0.001, respectively). Moreover, there was no significant difference in leaf area among different latitude areas (*P =* 0.614) (**Table 2**).

**Table 2:** The morphological traits of annul *D*. *involucrata* twig in three latitude regions (mean ± *SE*).

Traits	Population			df	F	*P*
	PW	TQ	GX			
Stem diameter (mm)	3.79 ± 0.10c	5.09 ± 0.12b	5.90 ± 0.28a	2	43.637	< 0.001***
Leaf area (cm²)	490.23 ± 40.75a	502.86 ± 42.65a	436.07 ± 46.68a	2	0.491	0.614 ns
Mean leaf area (cm²)	60.62 ± 3.58a	42.60 ± 2.21b	39.67 ± 3.79b	2	13.182	< 0.001***
Mean lamina length (cm)	10.33 ± 0.31a	8.33 ± 0.23b	7.56 ± 0.32b	2	23.538	< 0.001***
Mean lamina width (cm)	7.82 ± 0.26a	6.54 ± 0.18b	6.46 ± 0.35b	2	9.677	< 0.001***
Bract area (cm²)	153.31 ± 11.95b	130.32 ± 13.14b	205.96 ± 29.61a	2	4.569	0.014*
Flower stalk length (cm)	5.00 ± 0.18ab	4.47 ± 0.18b	5.68 ± .043a	2	5.786	0.005**
Flower stalk diameter (cm)	0.21 ± 0.01c	0.23 ± 0.01b	0.26 ± 0.01a	2	23.680	< 0.001***

Different lines letters in the same column meant significant difference (P<0.05). PW, Pingwu, n = 24; TQ, Tianquan, n = 30; GX, Gongxian, n = 14. The significance values of analyses are denoted as: ns, not significant; * P < 0.05, ** P < 0.01, and *** P < 0.001.

The biomass traits of reproductive, support and nutritive organ of *D. involucrata* twigs were significantly different in different latitude regions. Compared with the low- and middle-latitude regions, there were significantly lower twig mass, stem mass, flower mass and flower stalk mass in high-latitude region (*P* = 0.004 and < 0.001 and = 0.001 and < 0.001, respectively). The leaf mass and lamina mass was the minimum in the high-latitude region, which was significantly different from that in the middle-latitude region (*P* = 0.025 and = 0.019, respectively). Furthermore, the petiole mass and bract mass did not differ among different latitude regions (*P* = 0.16 and = 0.23, respectively) (**Table 3**).

**Table 3:** The biomass traits of annul *D*. *involucrata* twig in three latitude regions (mean ± *SE*).

Traits	Population			df	F	*P*
	PW	TQ	GX			
Twig mass (g)	3.52 ± 0.29b	5.45 ± 0.45a	5.17 ± 0.57a	2	6.176	0.004**
Stem mass (g)	0.90 ± 0.10b	1.94 ± 1.07a	2.04 ± 0.27a	2	12.962	< 0.001***
Leaf mass (g)	2.25 ± 0.19b	3.17 ± 0.28a	2.53 ± 0.25ab	2	3.901	0.025*
Lamina mass (g)	2.11 ± 0.18b	2.99 ± 0.26a	2.34 ± 0.23ab	2	4.204	0.019*
Petiole mass (g)	0.14 ± 0.01a	0.18 ± 0.02a	0.20 ± 0.02a	2	1.887	0.160 ns
Flower mass (g)	0.37 ± 0.03b	0.34 ± 0.04b	0.60 ± 0.09a	2	7.256	0.001**
Bract mass (g)	0.19 ± 0.01a	0.21 ± 0.02a	0.16 ± 0.02a	2	1.502	0.230 ns
Flower stalk mass (g)	0.03 ± 0.01b	0.03 ± 0.01b	0.07 ± 0.01a	2	25.053	< 0.001***

Different lines letters in the same column meant significant difference (P<0.05). PW, Pingwu, n = 24; TQ, Tianquan, n = 30; GX, Gongxian, n = 14. The significance values of analyses are denoted as: ns, not significant; * P < 0.05, ** P < 0.01, and *** P < 0.001.

### 3.2 Growth relationships among twig component organs in three latitude regions

**Figure 2:**
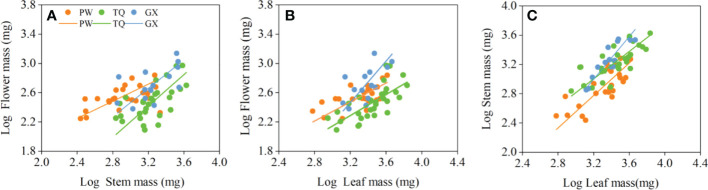
Growth relationships between flower mass and stem mass **(A)**, flower mass and leaf mass **(B)**, stem mass and leaf mass **(C)** in three latitude regions. PW, Pingwu; TQ, Tianquan; GX, Gongxian.

The flower mass was positively related to stem mass and leaf mass. The slopes between flower mass and stem mass, flower mass and leaf mass were significantly different in the three latitude regions (*P* = 0.019 and = 0.014, respectively), but there was no common slope for a regression between these traits. The slopes between flower mass and stem mass in low- and middle-latitude regions were 0.973 (95% CI = 0.598~1.585) and 1.075 (95% CI = 0.794~1.455), respectively. Moreover, the slopes between flower mass and leaf mass in low- and middle-latitude regions were 1.419 (95% CI = 0.881~2.285) and 1.009 (95% CI = 0.771~1.320), respectively, which indicated an isometric growth relationship. On the other hand, the slopes between flower mass and stem mass, flower mass and leaf mass in high-latitude region were 0.586 (95% CI = 0.431~0.797) and 0.650 (95% CI = 0.463~0.911), respectively, which suggested an allometric growth relationship, and the growth rate of flower mass was significantly lower than that of stem mass or leaf mass (**Table 4**; **Figures 2A, B**).

**Table 4:** Common slopes and SMA regression parameters for the relationships among the functional traits of *D*. *involucrata* twig in three latitude regions.

Y-X	SMA regression parameters						Common slopes		
	Site	R^2^	P(r)	Slope	Y-intercept	P(h)	Slope-c	CIs	P_(slop)_
Flower mass – Stem mass	PW	0.500	< 0.001***	0.586	0.847	0.019 ns	—	—	—
	TQ	0.368	< 0.001***	1.075	-1.015				
	GX	0.350	0.026*	0.973	-0.449				
Flower mass – Leaf mass	PW	0.390	< 0.001***	0.650	0.394	0.014 ns	—	—	—
	TQ	0.507	< 0.001***	1.009	-1.012				
	GX	0.381	< 0.001***	1.419	-2.070				
Stem mass – Leaf mass	PW	0.611	< 0.001***	1.109	-0.772	0.06 ns	1.130	0.943~1.324	0.196 ns
	TQ	0.574	< 0.001***	0.939	-0.003				
	GX	0.791	< 0.001***	1.458	-1.666				
Bract area – Flower stalk mass	PW	0.371	0.002**	1.046	0.669	0.988 ns	1.065	0.895~1.269	0.472 ns
	TQ	0.520	< 0.001***	1.084	0.577				
	GX	0.694	< 0.001***	1.055	0.365				
Bract area – Flower mass	PW	0.655	< 0.001***	1.227	-0.963	0.268 ns	1.090	0.947~1.243	0.232 ns
	TQ	0.707	< 0.001***	0.955	-0.295				
	GX	0.855	< 0.001***	1.147	-0.873				
Flower stalk mass – Flower mass	PW	0.813	< 0.001***	1.173	-1.560	0.129 ns	1.054	0.921~1.195	0.447 ns
	TQ	0.680	< 0.001***	0.881	-0.805				
	GX	0.831	< 0.001***	1.088	-1.173				
Petiole mass – Leaf area	PW	0.934	< 0.001***	1.192	-1.066	0.697 ns	1.151	1.073~1.235	<0.001***
	TQ	0.893	< 0.001***	1.122	-0.781				
	GX	0.953	< 0.001***	1.130	-0.698				
Petiole mass – Lamina mass	PW	0.918	< 0.001***	1.142	-1.651	0.350 ns	1.131	1.053~1.207	0.001**
	TQ	0.903	< 0.001***	1.056	-1.419				
	GX	0.971	< 0.001***	1.175	-1.675				
Petiole mass – Leaf mass	PW	0.928	< 0.001***	1.135	-1.661	0.387 ns	1.124	1.052~1.194	0.001**
	TQ	0.913	< 0.001***	1.055	-1.443				
	GX	0.976	< 0.001***	1.160	-1.666				

R^2^ represents the correlation coefficients between traits, P(r) represents the level of significance testing for relationship between traits, Slope represents the allometric growth slope between traits, y-intercept represents the intercept of y axial, P(h) represents the heterogeneity of slope, Slope-c represents the common slope, CIs represents the 95% confidence intervals, P_(slop)_ represents the level of significance testing for common slope. PW, Pingwu, n = 24; TQ, Tianquan, n = 30; GX, Gongxian, n = 14. The significance values of analyses are denoted as: ns, not significant; * P < 0.05, ** P < 0.01, and *** P < 0.001. Y-X represents the Y-axis and the X-axis, respectively.

In addition, stem mass was positively related to leaf mass in different latitude regions, with a common slope of 1.130 (95% CI = 0.943~1.324), indicating an isometric growth relationship (**Table 4**; **Figure 2C**). Furthermore, a significant shift was found between different latitude regions both in the y-intercept and along the common slope (*P* < 0.001 and < 0.001, respectively), suggesting high-latitude region had smaller stem mass for a given leaf mass than the low- and middle- latitude regions did (**Table 5**; **Figure 2C**).

**Table 5:** Tests for heterogeneity of slope, and shift in intercept for the relationships among functional traits of annual *D*. *involucrata* twig in three latitude regions.

Y-X	Shift along the common slope				Shift in elevation			
	PW	TQ	GX	P	PW	TQ	GX	P
Flower mass – Stem mass	—	—	—	—	—	—	—	—
Flower mass – Leaf mass	—	—	—	—	—	—	—	—
Stem mass – Leaf mass	6.621	7.135	7.067	<0.001***	-0.842	-0.656	-0.559	<0.001***
Bract area – Flower stalk mass	3.663	3.515	4.145	0.001**	0.642	0.603	0.347	<0.001***
Bract area – Flower mass	4.919	4.746	5.207	0.017*	-0.614	-0.627	-0.715	0.026*
Flower stalk mass – Flower mass	4.095	3.966	4.647	<0.001***	-1.258	-1.232	-1.081	<0.001***
Petiole mass – Leaf area	5.135	5.251	5.254	<0.001***	-0.957	-0.858	-0.753	0.643 ns
Petiole mass – Lamina mass	5.795	6.069	6.031	0.121 ns	-1.617	-1.676	-1.530	<0.001***
Petiole mass – Leaf mass	5.803	6.073	6.046	0.122 ns	-1.625	-1.681	-1.544	<0.001***

PW, Pingwu, n = 24; TQ, Tianquan, n = 30; GX, Gongxian, n = 14. The significance values of analyses are denoted as: ns, not significant; * P < 0.05, ** P < 0.01, and *** P < 0.001. Y-X represents the Y-axis and the X-axis, respectively.

### 3.3 Growth relationships among reproductive organ traits in three latitude regions

**Figure 3:**
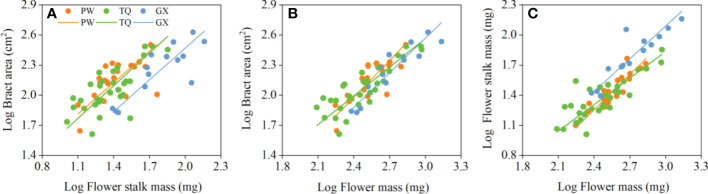
Growth relationships between bract area and flower stalk mass **(A)**, bract area and flower mass **(B)**, flower stalk mass and flower mass **(C)** in three latitude regions. PW, Pingwu; TQ, Tianquan; GX, Gongxian.

Bract area was significantly related with flower stalk mass and flower mass, and the common slopes for these relationships were 1.065 (95% CI = 0.895~1.269), and 1.090 (95% CI = 0.947~1.243), respectively (**Table 4**; **Figures 3A, B**), which results indicated an isometric growth relationship. Moreover, a significant shift was also found between different latitude regions in the y-intercept and along the common slope (*P* < 0.001 and = 0.026; *P* = 0.001 and = 0.017, respectively), suggesting low-latitude region had smaller bract area for a given flower stalk mass or flower mass than the middle- and high-latitude regions did (**Table 5**; **Figures 3A, B**).

In addition, the flower stalk mass was also closely related to the flower mass in different latitude regions, with a common slope of 1.054 (95% CI = 0.921~1.195), which indicated an isometric growth relationship (**Table 4**; **Figure 3C**). Moreover, a significant difference in elevation was found between different latitude regions in the y-intercept and along the common slope (*P* < 0.001 and < 0.001, respectively), suggesting that the low-latitude region had higher flower stalk mass for a given flower mass than the middle- and high-latitude regions did (**Table 5**; **Figure 3C**).

### 3.4 Growth relationships among vegetative organ traits in three latitude regions

**Figure 4:**
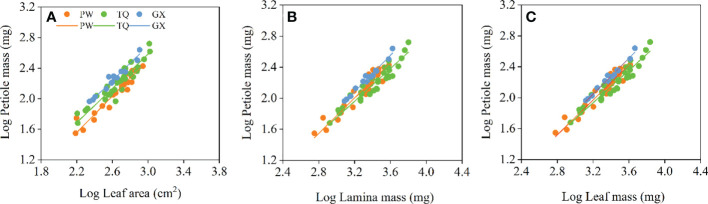
Growth relationships between petiole mass and leaf area **(A)**, petiole mass and lamina mass **(B)**, petiole mass and leaf mass **(C)** in three latitude regions. PW, Pingwu; TQ, Tianquan; GX, Gongxian.

Petiole mass was significantly related to leaf area, lamina mass and leaf mass, and the common slopes for these relationships were 1.151 (95%CI = 1.073~1.235), 1.131 (95%CI = 1.053~1.207) and 1.124 (95%CI = 0.990~1.000), respectively. These results indicated an allometric growth relationship and the growth rate of petiole mass was significantly higher than those of leaf area, lamina mass and leaf mass in different latitude regions (**Table 4**; **Figures 4A–C**). Furthermore, between different latitude regions, a significant shift was found along the common slope of the relationship of petiole mass and leaf area (*P* < 0.001). Meanwhile, a significant difference in elevation was found in the y-intercept of the growth relationship of petiole mass and lamina mass, petiole mass and leaf mass (*P* < 0.001 and < 0.001, respectively). There was significantly higher petiole mass per unit lamina mass or per unit leaf mass in low- latitude region than in middle- and high-latitude regions (**Table 5**; **Figures 4A–C**).

## 4 Discussion

Plants have the ability of plastic growth, and their morphological and physiological characteristics will change with environmental changes. In different habitats, plants may adopt the corresponding adaptive mechanism. For example, in high latitudes, *Pinus hwangshanensis* adjust the size of branches and leaves to adapt to the reduction of xylem vessel diameter caused by temperature reduction (Li et al., 2017), and *Lavandula latifolia* extend the length of labellum to cope with the low temperature environment (Herrera et al., 2006). Frenne et al. (2013) studied 98 species of plants worldwide and found that mean annual temperature and mean annual precipitation on the latitudinal gradient were the main factors affecting plant leaf traits. These studies demonstrated that environmental factors such as temperature and water content decrease as latitude increases, plants show some phenotypic adaptability to survive environmental changes. On this basis, we speculated that the functional traits and distribution strategies of *D. involucrata* twigs vary with latitudinal gradient. In this study, the individual size and biomass of *D*. *involucrata* twigs were the smallest in high-latitude region, with a trend of becoming smaller and shorter, which is consistent with previous studies (Kollmann and Bañuelos, 2004; Méndez-Alonzo et al., 2008; Moles et al., 2009). This phenomenon be explained that low-temperature in high-latitude region may constrain the photosynthesis of leaves, and reduce the absorption of carbon dioxide and the flow of important mineral elements in plants, thus restricting twig growth (Costa et al., 2017). Meanwhile, low temperature also inhibit the activities of metabolic enzymes, resulting in suppression of the related biochemical reactions and hence decrease in the growth rate (Reich and Oleksya, 2004). Furthermore, low temperature and water constraints may shorten the growing season of plants and prolong the dormancy period in winter. Long time of low temperature inhibits the enzymes rich in N and P in plants, the biochemical reaction rate of plants decreases and the growth rate slows down (Reich and Oleksya, 2004). As a result, the biomass of annual twigs in high-latitude region is lower than in low- and middle-latitude regions. On the other hand, as the supporting structure of the plant, stem has to bear the weights of branches and accessories like leaves, flowers and fruits to assure mechanical safety (Yang et al., 2010). Accordingly, the biomass of *D. involucrata* stem decreases due to the reduced biomass of leaves, flowers and other organs in high-latitude region. Therefore, the total resource acquisition of plants decreases with the increase in latitude, and the total resources allocated to different organs will decrease accordingly. However, we found some functional traits of *D. involucrata* twig did not change with latitudinal gradient. For example, with the increase of latitude, the mass of twig, leaf, lamina and bract first increased and then decreased, especially for leaf traits. This phenomenon may be caused by differences in temperature and water conditions among different latitude regions. Rainfall in the middle-latitude region is the most abundant among three *D. involucrata* population distribution regions, and water conditions are obviously better than the other two regions when the temperature is suitable. Hence, biomass will be more allocated to the leaves under good hydrothermal conditions, so as to improve the resource utilization efficiency by increasing the effective photosynthetic leaf area.

At the twig level, we found that investment in leaf and stem increased continuously although the total biomass of *D. involucrata* twig decreased with the increase of latitude. Flower mass, stem mass and leaf mass present isometric growth relationships in low- and middle-latitude regions, while the growth rate of flower mass in high-latitude region is significantly lower than that of stem mass and leaf mass. This demonstrates that *D. involucrata* twig increases nutrition and supports allocations in high-latitude region but decreases reproduction allocation. In high latitudes, low temperature causes damage to the photosynthetic carboxylation process of leaves (Frenne et al., 2013). Plants increase resource allocation to leaves, which is beneficial for light interception and carbon fixation. In this study, the mean leaf area of *D. involucrata* twig increased with increasing latitude, which also proved the increasing resource allocations to leaves. Moreover, the heat exchange capacity of large leaf edges is relatively weak, which is conducive to adapting to a low-temperature environment (Ackerly et al., 2002; Yang et al., 2008). In addition, the low-temperature environment in high-latitude region is easy to cause embolism of stem, resulting in low transmission efficiency and metabolic decomposition (Li et al., 2017). Thus, enhanced resource allocations to stem help plants to improve transportation efficiency and relieve metabolic stress. This is consistent with previous research conclusions that the risk of plant nutrient and water transport will increase with the increase in habitat stress, whereas such risks can be reduced by increasing resource allocation to stem (Sun et al., 2006; Xiang et al., 2009). Sun et al. (2006) studied 59 woody plants in temperate zones and found that the biomass allocated to stems was usually more at the level of twigs under a low temperature environment. Wright et al. (2002) studied the leaf traits of 2500 vascular plant species from 175 sites around the world, and found that leaves had a higher average leaf area under a low temperature environment. These results are consistent with our findings that a isometric growth relationship between flower mass and stem mass or between flower mass and leaf mass in low- and middle- latitude regions, but a allometric growth relationship (slope < 1) in high-latitude region to cope with lower temperature environmental. Therefore, increased nutrition and support allocations suggest that *D. involucrata* twig adapts to a high-latitude, cold environment by allocating the limited resources to leaf and stem and decreasing allocation to flower. This also reflects a trade-off of *D. involucrata* twigs in the investment of various organs to resist cold environment.

At the flowering level, there is an isometric relationship between the bract area and flower stalk mass. However, the variations in the common slope shows that the bract area and flower stalk mass of the population in low-latitude region are larger and higher than those in high-latitude region. Due to a more appropriate environment and many insect species in low-latitude region, a larger bract area can attract more pollinators, thereby increasing the probability of pollination (Harder and Johnson, 2009). Compared to the plants in low-latitude region, plants in high-latitude region have smaller and thicker bract, which can better protect the capitulum from low-temperature disturbances (Liu T. T. et al., 2020). In addition, as a support organ, the flower stalk has to bear the weights of two prominent bracts and the capitulum at the end. Plants *D. involucrata* in low-latitude region have a larger flower than other two regions, thus increasing the possibility of being attacked by strong wind and rain, means that the mechanical force on the flower stalk increases correspondingly. According to Niklas (1999), large plants usually have high reproduce cost, which requires them to invest more resources to strengthen mechanical support and protect flower organs from bad climates. In this study, we found that flower stalk mass in low-latitude region was more than in high-latitude region for a given flower mass. This also proved the increased resource allocation to flower stalks in low-latitude region. Therefore, *D. involucrata* tend to develop larger bract and stronger flower stalk in low-latitude region but a smaller bract to cope with low-temperature environmental stress.

At the leaf level, there was an allometric growth relationship between lamina and petiole in different latitude regions, with petiole having higher biomass growth rate than that of lamina. This result is consistent with previous studies (Niinemets et al., 2006; Niinemets et al., 2007; Li et al., 2008). Li et al. (2008) also found that in all species, there was an allometric relationship with a slope < 1.0 between leaf mass or area and petiole mass. This indicates that the increase of blade investment cannot keep up with the increase of blade support structure investment, which is a mode of diminishing returns (Shi et al., 2020b; Guo et al., 2021; Li et al., 2022). Petiole has to not only transport water and nutrients to leaves but also support the static weight of leaves and resist dynamic tensile forces, such as winds and rains (Li et al., 2008). These require extra biomass resource allocation to the petiole. Hence, there is an allometric relationship between leaves and petiole rather than an isometric one. In this study, the petiole mass in low- and middle-latitude regions is higher than that in high-latitude region for a given lamina mass, indicating a higher resource allocation to supports. The reason is that the petiole has to bear more weight in low- and middle-latitude regions than in high-latitude region, which is attributed to the higher lamina mass. On the other hand, it has been mentioned in the above study that the growing season of plants shortens, and the dormancy period prolongs in the high-latitude region due to the decreased temperature. Compared with high-latitude region, *D. involucrata* leaves in low- and middle-latitude regions germinate earlier but fall later. Increasing resource allocation to petiole is to prepare for early germination of new leaves, and delivery of water and nutrients to leaves that fall later. This is consistent with the finding of Li et al. (2008) that temperate evergreen broad-leaved tree species have higher investment in petioles than deciduous species. In order to keep consistent with leaves, petioles have to increase their resistance to freezing blockage by increasing more investment (Cavender-Bares et al., 2005). Therefore, we concluded that increasing resource allocation to petiole in low- or middle-latitude region represents an adaptive response of *D. involucrata* twig to a relative temperate environment.

Furthermore, according to the future prediction of climate models, the change of global climate may lead to the reduction of species distribution and the serious loss of biodiversity (Pearson and Dawson, 2003; Thomas et al., 2004; Rı´o and Penas, 2006). *D. involucrata* is highly sensitive to environmental factors such as temperature seasonality, precipitation in the hottest seasons and mean annual temperature. Meanwhile, the habitat of *D. involucrata* is affected by human activities and increased land use, which result in the narrowing of its distribution range. According to the findings of Tang et al. (2017), the projected decline in potential habitat area by 2070 under global climate change, which indicate that the habitat of *D. involucrata* will become very vulnerable. In view of the influence of climate change on the geographic distribution of *D*. *involucrata*, we suggest that undisturbed wild *D*. *involucrata* communities should be protected firstly, and build nature reserves, breeding bases and monitoring sites in the existing habitats of *D*. *involucrata*.

In conclusion, this study confirmed that *D. involucrata* adjusted the resource allocation and reproduction strategy with the change of latitude. The morphology and biomass of *D. involucrata* twig are negatively related to latitude, and resource is invested more to leaf and stem but less to flower in high-latitude region. Our results suggest that *D. involucrata* adapts to heterogeneous environments by changing the phenotypic plasticity and the relationship of trade-off of the investment-benefit-cost among traits to achieve population reproduction. These results can provide valuable information for the protection of the rare plant *D. involucrata*. Furthermore, there exist some disadvantages in our study that only three populations along latitude were selected, and the response of branchlet mechanical strength, leaf element content to latitude were ignored, which may have potential limitations to our results. More research work needs to be carried out from these perspectives in the future.

## Data availability statement

The original contributions presented in the study are included in the article/supplementary material. Further inquiries can be directed to the corresponding authors.

## Author contributions

XX conceived and designed the study. ZL, TL, XC, WX, TD and QL performed the experimental work. ZL analyzed the data and wrote the paper. All authors contributed to the article and approved the submitted version.
